# A Strategy for Origins of Life Research

**DOI:** 10.1089/ast.2015.1113

**Published:** 2015-12-01

**Authors:** Caleb Scharf, Nathaniel Virgo, H. James Cleaves, Masashi Aono, Nathanael Aubert-Kato, Arsev Aydinoglu, Ana Barahona, Laura M. Barge, Steven A. Benner, Martin Biehl, Ramon Brasser, Christopher J. Butch, Kuhan Chandru, Leroy Cronin, Sebastian Danielache, Jakob Fischer, John Hernlund, Piet Hut, Takashi Ikegami, Jun Kimura, Kensei Kobayashi, Carlos Mariscal, Shawn McGlynn, Brice Menard, Norman Packard, Robert Pascal, Juli Pereto, Sudha Rajamani, Lana Sinapayen, Eric Smith, Christopher Switzer, Ken Takai, Feng Tian, Yuichiro Ueno, Mary Voytek, Olaf Witkowski, Hikaru Yabuta

**Affiliations:** ^1^ Columbia University, New York, New York, USA.; ^2^ Earth-Life Science Institute (ELSI), Tokyo Institute of Technology, Tokyo, Japan.; ^3^ Institute for Advanced Study (IAS), Princeton, New Jersey, USA.; ^4^ University of Ochanomizu, Tokyo, Japan.; ^5^ Middle East Technical University, Ankara, Turkey.; ^6^ Universidad Nacional Autónoma de México, Mexico City, Mexico.; ^7^ NASA Jet Propulsion Laboratory, California Institute of Technology, Pasadena, California, USA.; ^8^ Foundation for Applied Molecular Evolution, Gainesville, Florida, USA.; ^9^ University of Hertfordshire, Hertfordshire, UK.; ^10^ University of Tokyo, Tokyo, Japan.; ^11^ ELSI Origins Network (EON), Earth-Life Science Institute (ELSI), Tokyo Institute of Technology, Tokyo, Japan, and Emory University, Atlanta, Georgia, USA.; ^12^ University of Glasgow, Glasgow, UK.; ^13^ Sophia University, Tokyo, Japan.; ^14^ Yokohama National University, Yokohama, Japan.; ^15^ Dalhousie University, Halifax, Canada.; ^16^ Tokyo Metropolitan University, Tokyo, Japan.; ^17^ Johns Hopkins University, Baltimore, Maryland, USA.; ^18^ ProtoLife.; ^19^ CNRS—University of Montpellier, Montpellier, France.; ^20^ University of Valencia, Valencia, Spain.; ^21^ Indian Institute of Science Education and Research (IISER), Pune, India.; ^22^ University of California, Riverside, California, USA.; ^23^ Japan Agency for Marine-Earth Science and Technology (JAMSTEC), Yokosuka, Japan.; ^24^ Center for Earth System Science, Tsinghua University, Beijing, China.; ^25^ NASA Astrobiology Program, Washington, DC, USA.; ^26^ Osaka University, Osaka, Japan.; ^27^ Friedrich Schiller University, Jena, Germany.

## Abstract

**Contents**

1. Introduction

1.1. A workshop and this document

1.2. Framing origins of life science

1.2.1. What do we mean by the origins of life (OoL)?

1.2.2. Defining life

1.2.3. How should we characterize approaches to OoL science?

1.2.4. One path to life or many?

2. A Strategy for Origins of Life Research

2.1. Outcomes—key questions and investigations

2.1.1. Domain 1: Theory

2.1.2. Domain 2: Practice

2.1.3. Domain 3: Process

2.1.4. Domain 4: Future studies

2.2. EON Roadmap

2.3. Relationship to NASA Astrobiology Roadmap and Strategy documents and the European AstRoMap

Appendix I

Appendix II

Supplementary Materials

References

## 1. Introduction

### 1.1. A workshop and this document

A workshop was held August 26–28, 2015, by the Earth-Life Science Institute (ELSI) Origins Network (EON, see Appendix I) at the Tokyo Institute of Technology. This meeting gathered a diverse group of around 40 scholars researching the origins of life (OoL) from various perspectives with the intent to find common ground, identify key questions and investigations for progress, and guide EON by suggesting a roadmap of activities.

Specific challenges that the attendees were encouraged to address included the following: What key questions, ideas, and investigations should the OoL research community address in the near and long term? How can this community better organize itself and prioritize its efforts? What roles can particular subfields play, and what can ELSI and EON do to facilitate research progress? (See also Appendix II.)

The present document is a product of that workshop; a white paper that serves as a record of the discussion that took place and a guide and stimulus to the solution of the most urgent and important issues in the study of the OoL. This paper is not intended to be comprehensive or a balanced representation of the opinions of the entire OoL research community. It *is* intended to present a number of important position statements that contain many aspirational goals and suggestions as to how progress can be made in understanding the OoL.

The key role played in the field by current societies and recurring meetings over the past many decades is fully acknowledged, including the International Society for the Study of the Origin of Life (ISSOL) and its official journal *Origins of Life and Evolution of Biospheres*, as well as the International Society for Artificial Life (ISAL).

### 1.2. Framing origins of life science

H. James Cleaves II (ELSI), Caleb Scharf (Columbia University), Nathaniel Virgo (ELSI)

#### 1.2.1. What do we mean by the origins of life (OoL)?

Since the early 20^th^ century the phrase OoL has been used to refer to the events that occurred during the transition from nonliving to living systems on Earth, i.e., the origin of terrestrial biology (Oparin, 1924; Haldane, 1929). The term has largely replaced earlier concepts such as abiogenesis (Kamminga, 1980; Fry, 2000).

The historical development of OoL science was dominated by geology (*e.g.*, as the age of Earth and the nature of its surface environment at the time of the OoL are central to understanding the problem), paleontology (the earliest evidence for life offers constraints on the timing of the OoL and potentially the earliest niches), and comparative biology (in that common ancestral biological properties may be inferred).

However, OoL questions have also driven experimental sciences. These include chemistry (allowing potential steps in the process of the OoL to be directly tested in the laboratory) and molecular biology (as it contributes to phylogenetic reconstructions and more recently to experimental studies of putative “RNA World” chemistry) as principal areas of inquiry. These areas have in turn generated many new questions, for example the “Paradoxes” or “Open Questions” in the OoL (Benner, 2014; Luisi and Kuruma, 2014).

Origins of life science has also grown to encompass what is assumed to be possible in other planetary contexts, opening up the realm of cosmic OoL, where fields like planetary science and astronomy can provide insight.

The origin of life has also been a long-established topic in theoretical and evolutionary biology and theoretical chemistry. Within these fields, topics such as the minimal fidelity of replication required for sustained evolution (Eigen and Schuster, 1978) or the encapsulation of metabolic reactions within membranes (Gánti, 2003) can be addressed somewhat independently of any specific molecular context.

Imperfect reproduction, replication, or division (which are subtly different processes) are all related to the possibility of population growth and the natural selection and evolution of living systems. For many researchers in systems chemistry, these processes are the touchstones that readily capture the nature of life and its emergence. In this context, the OoL can be considered as a continuous, gradual process starting from chemical autocatalysis or chemical replication and then complexifying into more sophisticated forms of replication.

Recently (Pascal and Pross, 2015), an attempt was made to reconcile Darwinian theory and the second law of thermodynamics within a unifying framework through the concept of persistence. This approach applies to regular systems as a drive toward increased probability (entropy). Provided they are held far from equilibrium (*i.e.*, fed with energy), systems that are capable of making more of themselves can evolve toward increased kinetic stability, which is another form of persistence.

Questions conceptually (and critically) related to the OoL have also been found in other domains. As physics progressed over the 20^th^ century, new puzzles arose regarding self-organization in non-equilibrium systems, with life being seen as one example of a more general phenomenon (Schrödinger, 1944; Maturana and Varela, 1980; Rosen, 1991). More recently, the spontaneous organization of life-like processes in non-natural milieu has become a domain of inquiry in its own right, for example, *in vitro* in the form of novel chemical systems or *in silico*, adding artificial life (A-Life) studies and computer science to the investigative repertoire.

A key outcome of this workshop (*see Outcomes below*) was a consensus that the interaction and integration of these many fields will be crucial for making significant progress in the years to come. Many of the discussions and commentaries that supplement this white paper detail how the various approaches to understanding the OoL may interrelate. These ideas have been further distilled into a proposed set of definitions for *types* of OoL and types of *approaches* to studying the OoL.

#### 1.2.2. Defining life

A definition of life is notoriously difficult, although many putative ones exist (Pályi *et al.*, 2002; Bedau and Cleland, 2010). By contrast, the concept of “origin” is much more rigid, generally connoting the point of inception of some phenomenon. Therefore, we suggest that what OoL studies all ultimately address is the *onset of the various organizational phenomena that we associate with the living world*. This unites all areas of research, from laboratory experimentation to Earth and planetary exploration, theory, and computation.

Nonetheless, while all fields that study the OoL ultimately study lifelike phenomena, the specific goals of each approach may be somewhat different. For example, the field of A-Life has been characterized as the study of “life as it could be” versus “life as it is” (Langton, 1989), with the aim of understanding biological phenomena (and sometimes their origins) on an abstract, generalizable level.

In contrast, approaches rooted in biochemistry or geochemistry are often concerned with much more detailed puzzles regarding molecular mechanisms that were at play on early Earth. We will see throughout this paper and its supplementary material that these sets of questions are not as neatly separable as traditional disciplinary boundaries may make them appear.

With respect to the OoL on Earth, investigations have expanded over the years to encompass a great deal of “ancillary” information, including prebiological geochemical conditions and post-OoL biological evolution and ecology. Many of these avenues of research are capable of giving insights to the core phenomena of the OoL. We also note that from the standpoint of any of these disciplines the central problem of the OoL is not yet resolved. Therefore, it may at present be counterproductive to too-hastily restrict or reject any seemingly oblique approaches.

Rather, we strongly recommend that OoL researchers seek to frame their efforts in the spirit of cooperation between multiple fields of inquiry, each driven by its own set of core questions and with its own approach to answering them.

Although this recommendation might appear self-evident, the suggested interdisciplinary collaboration is not always straightforward or obvious. Increasing the communication and understanding between the diverse existing approaches is a first step. The eventual goal must be to advance the entire OoL enterprise by enabling new insights, establishing common resources, and convincing funding agencies of the significance of OoL phenomena.

#### 1.2.3. How should we characterize approaches to OoL science?

Approaches to the OoL can be broadly divided into three classes, which can be termed *historical, synthetic,* and *universal.* This need not be taken as an absolute classification but rather as a bird's-eye characterization of the ways in which the central questions can be approached, with many studies falling into more than one category (*see Outcomes below*).

Historical approaches are characterized by research to determine the path of events that led to biology on Earth (and elsewhere, to the degree that such studies are generalizable). For historical approaches, success is typically judged by explaining evidence left in the geological record or in the nature of biochemistry, or by constructing narratives that are consistent with this evidence—typically constrained by presumed “plausible” prebiological environmental conditions and available reagents.

Synthetic approaches are less concerned with how life arose historically and more with how to create the process *de novo*, either in simulation or in the laboratory. Success is measured in terms of being able to create a system with some desired set of properties, even if it does not resemble biological life in every respect. Synthetic approaches are not always concerned with prebiotic plausibility and thus can aim for something that differs markedly from “real” or “modern” biology in terms of composition. This includes much of the work in A-Life, as well as attempts to create chemically orthogonal “living” systems *in vitro* or in unicellular contexts. We offer a more fine-grained classification of synthetic approaches in the *Outcomes* below.

Finally, universal approaches are concerned primarily with questions about necessary and sufficient conditions: can life emerge on planets quite different from Earth, or even in simulated “universes” with quite different “physics” from ours? Are there deep theoretical principles through which central processes in the OoL can be understood, irrespective of the domain in which they occur? This category includes aspects of astrobiology, A-Life, systems science, and evolutionary theory.

**FIG. 1.**
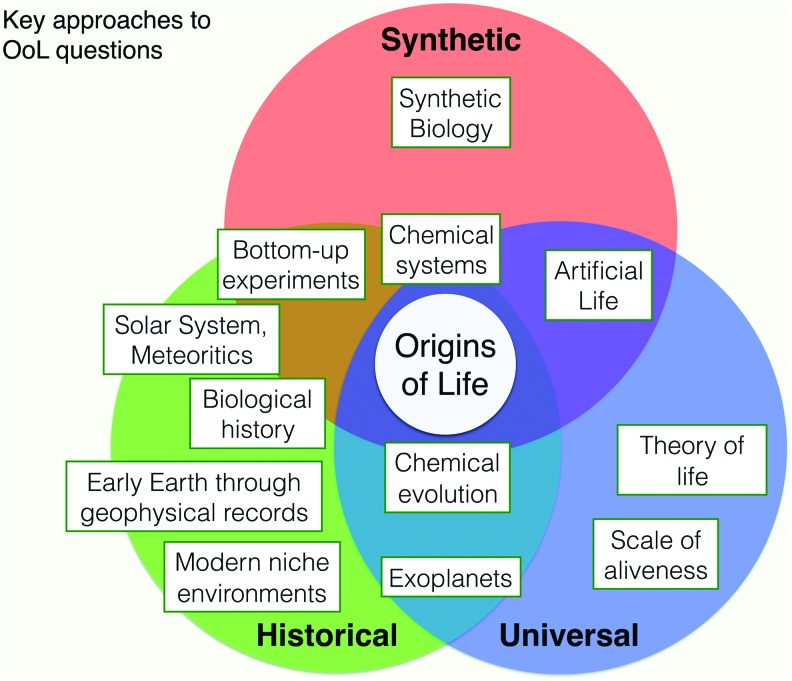
A concept map of the approaches to OoL science (Historical, Synthetic, and Universal) and where selected current areas of study sit in relation to each other and these three approaches. For example, the study of chemical systems overlaps with all three approaches, whereas the study of early Earth is primarily concerned with the single, Historical, nature of terrestrial OoL.

The concept map (Fig. 1) illustrates the relation and overlap of the principal variety of OoL investigative approaches detailed in this white paper to these proposed domains.

#### 1.2.4. One path to life or many?

The historical OoL on Earth remain almost entirely mysterious. Although there are a great many more-or-less detailed scenarios for how life may have originated on Earth, none has yet been generally accepted by a clear majority of the scientific community. Among other uncertainties, we do not currently know whether the path by which life arose on Earth is the only possible one in a natural context, or whether there are many other possibilities in alternate environments that could exist on other early Earth-like planets, or still others that might *only* be possible on other types of planetary bodies.

**FIG. 2.**
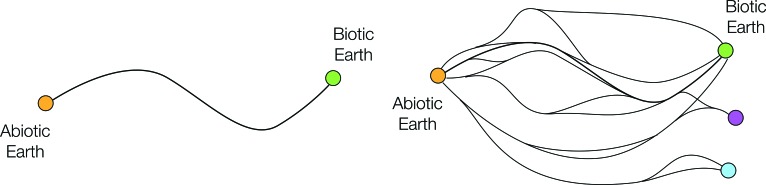
Left: A single historical path from an abiotic to a biotic planet. Right: Many possible paths leading to multiple end points, representing states that differ in some significant way from the biosphere we know on Earth.

Figure 2 illustrates two scenarios. At one extreme (left panel) there is only one possible sequence of events that could result in our planet developing a biosphere, implying that life could not emerge unless very specific conditions were present on Earth throughout its history.

By contrast, the right-hand diagram shows an alternative picture in which the same starting point—a prebiotic, Earth-like planet—can progress along multiple developmental paths. These converge onto multiple biological end states, with greater or lesser resemblance to Earth's modern biosphere. It is possible that life could originate on almost any sufficiently Earth-like planet, but the route through which this occurs could be wildly variable and thus extremely difficult to constrain.

We note that the frequency with which convergent evolution is observed in biology suggests that, at least after the emergence of Darwinian selection, there are often multiple paths to very similar end states. In this context, the crucial questions center on the relative ease with which life originates and the feedback between biology and global geochemical cycles.

On the other hand, observations of Mars and Venus suggest that a planet can fairly easily fail to be “Earth-like” enough to develop a long-lived surface biosphere, even if conditions early in the histories of those planets were conducive to the OoL. The key point here is that the space in between these two extremes is not currently well mapped.

**FIG. 3.**
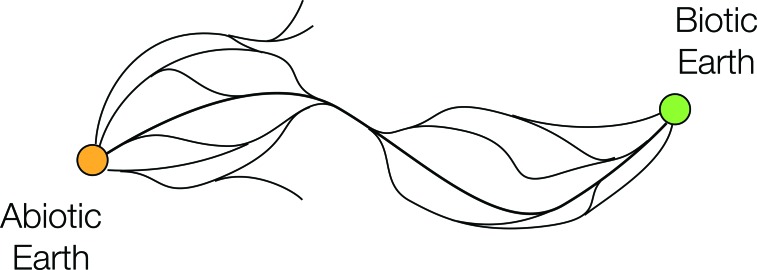
A bottleneck in the space of paths to life; some parts of the story are much more tightly constrained than others. In contrast to the case illustrated in Fig. 2 (right panel) where numerous “histories” lead to an OoL event, here only a specific “history” leads to an OoL event.

The sketch in Fig. 3 might be a more realistic representation than that shown in Fig. 2. Here, the planet's biotic development sometimes follows a multitude of branching and diverging paths, but at other times the paths pass through a bottleneck, that is, a specific sequence of events that must occur at the local or planetary scale for biological evolution to progress.

As an example from a later stage of evolution, pointed out by Maynard Smith and Szathmáry (1997): There seem to be many ways in which multicellularity can arise, as evidenced by its multiple origins on Earth, but the origin of eukaryotes may have happened only once and may have required a very specific set of circumstances to have produced the functional characteristics we observe today.

Historical contingency in biological evolution continues to be hotly debated and explored (*e.g.*, Blount *et al.*, 2008) and may indeed be informative for prebiological evolution. But we note that both random and deterministic processes are likely at play and that very rare or unlikely reaction pathways may nonetheless be deducible from first principles.

We suggest that the pathway maps sketched here (Figs. 2 and 3) can serve a number of critical purposes. First, they can form the basis of a useful tool for conceptual organization, inspiration, and communication between disciplines (*e.g.*, by clearly illustrating concepts such as bottlenecks, branching, and multiple OoL).

Second, we propose that an important task in understanding the historical OoL is to develop these cartoon sketches to produce a real, quantitative map of the pathways and constraints to abiogenesis. If some of the major bottlenecks could be definitively identified, this would represent significant progress. Even a more abstract proof-of-concept from a sufficiently complex simulation—providing an example of spreading emergence through one or more bottlenecks—could be very helpful.

Each type of approach to understanding the OoL has a role to play in this endeavor. For the historical, the geological record could in principle place strong constraints on the process for Earth. Such constraints may also hint at the frequency of occurrence of an origin of life on Earth-analog worlds. However, critical questions remain on the probability of life arising on worlds *unlike* Earth, or under experimental conditions or in abstract simulations that do not resemble any natural environment.

In places where a part of the story is not well understood, synthetic approaches can blaze new trails, demonstrating phenomena that may be analogous to processes in the natural environment. Universal approaches come to the fore where historical bottlenecks are not evident, helping elucidate cases where many possible processes can lead to the same end result.

We suggest also that mapping the unknown territory of the OoL in this way can help catalyze a shift in focus toward breaking down the problem into hypotheses that can be tested independently of one another. This process will in turn help foster better communication between disciplines and lead to faster progress in understanding OoL phenomena.

## 2. A Strategy for Origins of Life Research

### 2.1. Outcomes—key questions and investigations

(Based on a summarizing group discussion with contributions from all attendees, led by panel: Kensei Kobayashi, Leroy Cronin, Nathaniel Virgo, Christopher Switzer, Mary Voytek, Laura M. Barge, Robert Pascal, H. James Cleaves II.)

We have distilled the outcome of the specific discussions from the workshop into four domains: **Theory**, **Practice**, **Process**, and **Future Studies**. Within these divisions are sets of aspirational and actionable items that, if pursued, are proposed as a viable strategy for advancing understanding of the OoL in the near and mid term. As explained above, this distillation is not intended to be fully comprehensive. This listing represents a snapshot of opinion and discussion captured at a particular moment in the field's development. Additional opinions, perspectives, and reviews are catalogued in the Supplementary Material.

#### 2.1.1. Domain 1: Theory

**Theories and definitions of life.** The discussion at the workshop identified an urgent need for a better, comprehensive theory of life to better define the aims of OoL investigations, that is, to define the phenomena whose spontaneous onset is being studied. It was recognized that arguments or controversy over definitions, while not always helpful, may continue while we lack such a theory (*cf.* Cleland and Chyba, 2002). It was also noted that a compatible, substrate-neutral, quantitative theory of *evolution* is highly desirable.

Thus, our recommendation is an ongoing focus on the general development of theory, rather than on the widespread adoption of any particular existing framework. Previously proposed frameworks, such as autopoiesis or the chemoton, have nonetheless provided useful and fundamental insights.

We also note that a theory of life may be within reach (via advances in synthetic biology, A-Life, etc.) while a theory of the OoL still requires fundamental, perhaps revolutionary, developments.

It was generally agreed that progress will require treating life in terms of process and as a system. These approaches are closely aligned to the abstractions studied in the field of A-Life, such as dynamical systems, as well as those that have been explored previously in systems science and cybernetics.

Progress toward a theory of life may require substantial prior and parallel experimental work to better quantify the nature of relevant systems. There was broad group consensus that chemical systems are of particular interest here, not only because real biological systems are chemical in nature but because the nature of chemistry may present a unique substrate for the instantiation of OoL phenomena.

Many facets of OoL science are extraordinarily complex scientific or technological problems in their own right. For example, while scientists now have relatively simple and robust tools for studying the emergence and frequency of RNA-based catalysts from very large sets of random polymers, such technologies are not yet as widely available for peptides or other informational molecules besides the biological ones. The investigation and solution of these problems may require augmentation with tools such as “computer-assisted thinking” to help with abstraction and concept extraction (the reduction of a problem or phenomenon to a set of essential characteristics).

Even a general theory of the *origins* of life may not allow a full explanation of the specifics of terrestrial OoL, due to the historical nature of the question. But substantial progress should be possible through exploring and understanding the emergence of synthetic or A-Life and an enhanced dialogue between researchers in traditionally non-aligned fields. We note that there are significant ongoing efforts, including those focused on a re-conceptualization of the OoL (https://carnegiescience.edu/events/lectures/re-conceptualizing-origin-life), growing out of work on modeling OoL (*e.g.*, the MOL collaboration, https://github.com/ModelingOriginsofLife/March2014WhitePaper/wiki).

**An explicit acknowledgement, and classification, of the** ***types*** **of OoL and the types of** ***approaches*** **to understanding OoL**. Origins of life studies and dialogues often conflate the characteristics of origins events under consideration (*see Framing section above*). This can hinder communication within fields and is problematic for broader cross-disciplinary interactions.

In the introductory section, we suggested the terms *Historical, Synthetic,* and *Universal* as a broad classification of *approaches* to the OoL problem (Fig. 1). Current trends in all three approaches suggest the possibility of life based on a different set of molecular compounds from modern biology. We propose the development of a common language to describe the aims of origins studies, not just in terms of approach but in terms of the “type of life” that is the target of study.

Rather than trying to offer an exhaustive classification scheme, we offer the following tentative glossary of terms regarding the “types of life” considered in OoL studies. This list is suggested as the seed for a common terminology by which the relationships between approaches can be better understood. The terms below are not intended to be mutually exclusive.

*Terrestrial/Actual*—the OoL as they actually occurred on Earth; that is, the specific chain or sequence of events and mechanisms that led to the last universal common ancestor (LUCA) and early evolution soon after its appearance. Constraining our knowledge of the terrestrial OoL is the primary aim of many, but not all, approaches to the OoL.

*Extraterrestrial*—the OoL as they may have played out in environments beyond Earth. The life that results might be close to that of Earth or radically different.

*Nonstandard composition*—we introduce this as a catchall term to refer to life based on molecular components different from the ones we are familiar with in terrestrial biology. This idea has recently been gaining in popularity in both historical and synthetic approaches. See for example the contribution by L. Cronin in the Supplementary Material.

*Nonstandard structure*—we introduce this term to refer to life based on *similar* molecular components to extant Earth biology but put together in unfamiliar ways.

*Plausible*—in the absence of specific knowledge about the actual historical origin of Earth life, we often consider detailed scenarios for how the origin of life *might* have occurred, constrained by what is known about Earth's geological history and physical chemistry. This has been a common approach throughout much of the field's history.

*Reinvented*—not all approaches are constrained by plausibility. Instead, some deliberately target processes different from those that occurred on Earth to better understand the universal aspects of biology and its origin.

*In silico/Abstract*—it is also possible to consider the origin of life in more abstract terms. When computer simulation is the main methodology, this approach is often referred to as *in silico*. A great deal of work in A-Life is of this form.

While some of these terms are in common use, we draw particular attention to those that are not. *“Nonstandard”* approaches remain a fertile area for origins research. This is true within historical approaches (*e.g.*, Joyce *et al.*, 1987) as well as in more synthetic approaches. The “reinvention” of life in new, nonstandard forms will likely be beneficial to all origins research, insomuch as it demonstrates the wider tool kit with which lifelike systems can be constructed.

**An evaluation of the degree of completeness of any eventual OoL theory**. If history is any guide, standards for what would count as a complete OoL theory may be unlikely to be determined by scientific consensus in the near term. A more realistic goal is to try to assess the degree of completeness of any particular theory, whether about the OoL on Earth or elsewhere in the Universe.

Even there, it may be difficult to attain consensus. It will always be useful to examine OoL theories in comparison with their historical antecedents to evaluate their degree of completeness and how the evaluation of OoL models changes over time. A scrutiny of the historical development of such models, from a number of disciplinary backgrounds, will help highlight the different ways in which the OoL problem has been framed over time.

**A mapping abstraction of OoL**. A simple graphical representation of the fundamental trajectories and interactions of OoL pathways can be used as a common tool to summarize concepts and to illuminate where different approaches contribute and face similar challenges (*see Framing section*).

**FIG. 4.**
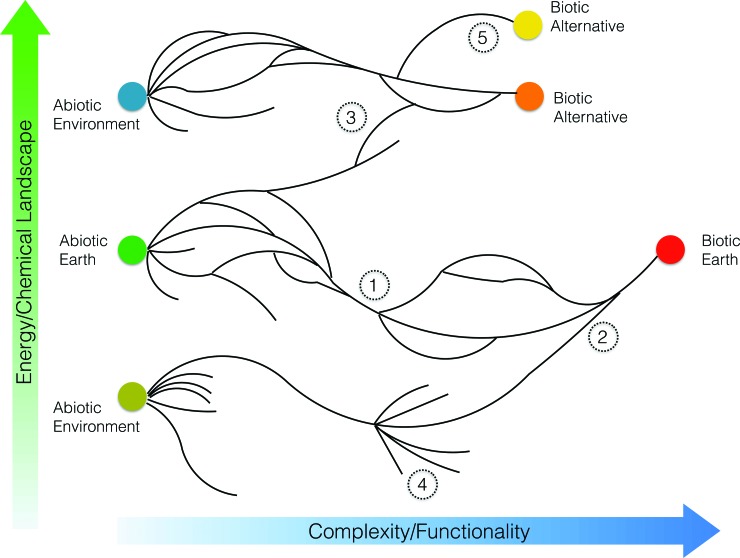
A conceptual representation of hypothetical pathways from abiotic to living states. An unspecified measure of complexity and/or functionality increases from left to right, and an unspecified measure of the energy or chemical landscape increases bottom to top. Labeled points illustrate various hypothetical situations: (1) A bottleneck—all histories must pass through here for terrestrial OoL—this therefore represents a critical focus for geological study or exploration (*e.g.*, Mars), *cf.* Fig. 2. (2) An alternate (nonterrestrial/actual) abiotic environment nonetheless leads to an exact match to terrestrial OoL. (3) A terrestrial abiotic landscape eventually leads to a nonterrestrial (alternate) biotic system of lower complexity. (4) A pathway exhibits rapid diversification of preliving systems (*e.g.*, molecular structures) although only one leads to an OoL event. (5) A nonterrestrial pathway splits at advanced complexity and leads to a separate OoL event within the same abiotic environment.

Branches, nodes, and bottlenecks can be related to complexity, information, chemical possibilities, emergent systemic properties, energy/fitness landscapes, and exploration priorities (*e.g.*, studies of extraterrestrial solar system environments). A further example is sketched here (Figure 4).

However, a consistent terminology is needed to make the best use of abstractions and conceptual representations and to avoid misunderstanding across fields (*e.g.*, “speciation” in biology has very different meaning than it does in chemistry). Thus, a cross-disciplinary translation process will be necessary.

Conceptual maps are a way to help express concepts that describe what is likely to be universal about life, as process and operation, from “wet” systems to “dry” A-Life. It may also help identify universal bottlenecks or steps that any terrestrial, plausible, or artificial OoL trajectory must pass through and are therefore strategically important areas of research.

**The need for a quantitative scale of living systems**. Although it has presently evaded us, it is clear that there is a critical need for a quantitative (even if incomplete) continuous scale or measurement that can be used as a practical tool to evaluate the “aliveness” of any given system.

There are a number of traits that typify life, such as complexity, adaptiveness, and thermodynamic disequilibrium. To develop a scale of “aliveness” will require a proper study of these and perhaps other variables, as well as the concept of “continuity” within these scales as they may apply to life.

Much like biological evolutionary processes in general, the transitions between abiotic and biotic systems or stable and metastable states (*i.e.*, states either sensitive to perturbation or that change very slowly) could be smooth and/or discontinuous.

The quantification of complexity is an important challenge since complexity is found at many different levels of biological organization, from subcellular levels up to ecosystems. Any such scale may be refined by experiment and comparison with other proposed scales.

Invoking the triviality or lack thereof of any object may help us frame how life works. Complex, nonliving objects, though made by living systems, could act as useful guides. The sand on a beach is clearly nonliving, but sand fired into a glass with a handle and other features was clearly made by a living system. Furthermore, a working scale could also be applied to evaluate the *environments* that support living systems.

Practical tools will be required to implement these scales. Various accepted techniques are used to detect, evaluate, and quantify living systems on Earth, and these technologies have already been adapted for use on other planets (*e.g.*, Mars). To a large extent, these techniques remain extremely terrestrial biology–centric, and it may be necessary to develop new technologies for life detection for both solar system exploration and for use in the laboratory (*e.g.*, wet A-Life). These methods may involve the quantification of differential responses of systems to varying conditions, the detection of novel chemical signals, or both.

A quantitative scale also points toward a need to develop a precise operational definition of life. A bottom-up approach might involve searching for the minimal set of molecules necessary to construct life *in vitro*. The laboratory study of simple autocatalytic chemical systems (for example, the formose reaction) may offer clues.

#### 2.1.2. Domain 2: Practice

**The further development of machine-chemistry-algorithm investigations**. Effective exploration of OoL scenarios will likely require the use of high-throughput chemical laboratory automation (*e.g.*, robotics, microfluidics), so-called “cyber chemistry.”

“Cyber-chemistry” offers new opportunities for exploring OoL landscapes and may strengthen ties between the wet and dry A-Life communities through the study of system dynamics and processes.

There is a need for experiments designed to identify the prebiotic selection of efficient self-reproducing *systems* instead of individual biomolecules. Cyber-chemistry may offer a method for accomplishing this.

**Learning about the robustness of living systems by mimicry**. Technology that has become ubiquitous for humans (*e.g.*, algorithms linked to real-world robotics or human-interaction systems, from industrial systems to predictive search engines) may capture some properties of living systems (*e.g.*, fitness and adaptation, autonomy) that could provide deep insight to emergence and complexity in OoL scenarios.

We should consider the application of these interactive technologies (and examine their inadvertent application, *e.g.*, the propagation of information and behavior via social media) to mimic living systems. This may represent a unique opportunity to identify signposts toward the abstraction of organisms.

**Long-term experiments**. The community should identify existing long-term experiments or potential experiments (*e.g.*, those spanning decades, centuries, or longer that are intentional or unintentional) and actively encourage the performance of new ones.

The challenge of studying OoL mechanisms that are potentially very slow, or of very low probability, has not been addressed in proper detail. This may be a serious problem for studying the OoL and is worthy of attention.

**Organized competitions as drivers of discovery and community**. Origins of life research should seek innovative and disruptive approaches to theory, experiment, and exploration that can serve to pull together traditionally isolated communities. Barriers to overcome include those of basic terminology and conceptual differences, even though common phenomena are under discussion.

Competitions, along the lines of competitions already held for artificial intelligence research (*e.g.*, the Turing Prize), autonomous vehicles (*e.g.,* the DARPA Grand Challenge), space exploration (*e.g.*, the X-Prize), and the like, could be useful for making progress in understanding the OoL—both in terms of channeling science activities and in terms of building community. Establishing safety protocols (*e.g.*, techniques to isolate successful, invasive living systems from the current natural or *in silico* environment) would be advisable and also a useful community exercise.

Specific suggestions include a challenge to bring the most “alive” system to the table, preferably via a multidisciplinary team effort, for example, by melding chemistry, A-Life, and robotics. Another concrete example could be a competition in the CRitical Assessment of Artificial Cellularity (CRAAC). The purpose of this would be for teams to enter their best “artificial cell.”

#### 2.1.3. Domain 3: Process

**ELSI/EON can be a critical safe harbor**. Research into the OoL appears to have reached a critical juncture, where it can flourish with greater integration into the broader scientific community and even play a leadership role. But to do so it needs help that augments long-standing efforts by (for example) ISSOL and other groups.

The Earth-Life Science Institute and EON can, and should, serve as “safe harbors” for scientists and projects in OoL that have traditionally remained on the periphery of many disciplines or have struggled for resources to make progress. Encouragement is needed to get people to move from established fields to carry out OoL research.

The field of astrobiology already overlaps greatly with a number of OoL areas (*e.g.*, early Earth, chemical systems in extreme environments, exploration, and the search for life), and many researchers are active in both the astrobiology and OoL communities and linked through, for example, NASA's support and interests in astrobiology. This is a connection that can be reinforced.

The Earth-Life Science Institute represents an unprecedented network of disciplines and a pool of expertise to draw on in a single institution—spanning earth sciences, chemistry, biology, astronomy, and computation. Structuring a set of OoL science modules in an open systems science approach (a group of parts creating a growing, renewing whole) will help advance the field. Support for ELSI beyond its initial 10-year period will be critical.

**The need to build dialogue between various subcommunities**. The origin of life is a problem that is studied by scientists working in a wide variety of fields. These communities differ not only in the types of questions they ask but also in what is taken to constitute an answer. Communication between such different perspectives can be difficult, but the origin of life is a problem that requires precisely this.

Questions raised at the workshop ranged from “what was the composition of the primitive atmosphere?” to “how can we formalize the notion of agency?” These questions might seem so different that there is little hope that either could be of any use in answering the other. Yet each question in its own way is motivated by a desire to know how biology can arise from nonbiology, and each in its way requires an answer if the origin of life on Earth is to be fully understood. Other questions, such as those addressing the emergence of metabolic networks, are asked by multiple fields but approached in such different ways as to seem incompatible.

Perhaps the greatest such divide is between theorists and experimentalists, across all disciplines. We believe that a great deal of progress can be made by training a new generation of theorists in OoL to work in tandem with experimentalists. This may result both in the generation of new theories that are more tightly constrained by empirical knowledge and in a greater sense of direction for experimental work.

Another important gap, which could easily be closed, is that between A-Life (or rather, the small subset of it that is concerned with the OoL) and the “traditional” OoL community. A-Life has traditionally been concerned with understanding life and its origins at the organism level rather than the biochemical level. An increase in cross-pollination of both ideas and questions could greatly benefit both the A-Life and OoL communities. ELSI/EON is in a good position to facilitate this by bringing members of both communities together and starting this dialogue.

There is a consensus that a full review of the work that A-Life has contributed to the OoL problem would be a positive contribution to this process of community dialogue. How this is initiated is, however, an open question.

Increasing communication between more traditional disciplines (chemistry, biology, biochemistry, mathematics, physics) on the particular topic of the OoL is also a priority.

In concert, attention needs to be paid to ensuring open access to data and data management, to constructing sophisticated metrics of community and group progress, and to understanding the history of the field and developing the philosophy of the field.

#### 2.1.4. Domain 4: Future studies

**Investigations targeting OoL-planetary-cosmos connections**. There is a need for continued and expanded investigation of the specifics of the nature of the early Earth environment, and for solar system and exoplanetary exploration in the context of the OoL, which are largely driven by notions derived from terrestrial/historical OoL science.

Important examples include the nature of Hadean Earth's (or Noachian Mars') atmospheric and oceanic chemistry, and the existence and partitioning of wet and dry environments in relation to organic compound synthesis, concentration, and reaction. These investigations should include the study of niche environments where unusual chemical and physical processes of potential importance for the OoL may have occurred.

As observational technology improves, exoplanets present an opportunity to study young terrestrial worlds as proxies for young Earth, both in terms of climate states and atmospheric chemistries. Other solar system bodies, for example Titan, Europa, and Enceladus, could also provide important observational data about chemical evolution.

Despite decades of study, a complete understanding of organics in meteorites and their role on Hadean Earth is still lacking, but critical. Equally, there is a need to understand how simple monomers can lead to more complex molecules in a variety of solar system settings and cosmic radiation environments (*e.g.*, interstellar space, comets, icy moons).

**Metabolic pathways, energy, and biology**. The investigation of metabolic pathways and their history before LUCA and before enzymes should be pursued.

What were the first energy sources (disequilibria) for life, and how were they used? Earth is a type of environment with many exploitable energy sources, and it may have been that early life was using one of the same sources that we can find life using today. It is important to continue to identify and enumerate sources of energy that are used by modern biological systems so that we understand how life works today. Mechanistic information on the use of energy sources is extremely helpful and may provide insight to OoL properties. Alternative energy sources, redox couples, and mechanistic pathways for their utilization should be investigated as possibilities both today and in the past.

What were the mechanisms of energy conservation (conversion) used by early life to organize material through the dissipation of disequilibria? Today, three mechanisms of energy conservation are recognized, which allow coupling of chemical reactions to metabolic “work.” They are (1) substrate-level phosphorylation, (2) charge separation across a membrane with ion pumps (aka, chemiosmosis), and (3) electron pair bifurcation.

An immediate challenge is to identify which of these mechanisms were operative early on and which may not have been. Each of these three are somewhat troubling in their own unique ways. For example, substrate-level phosphorylation allows only “direct” exchange of chemical energy and likely cannot function in low-energy environments since it cannot function in “ratchet” form. And while it is widely thought that chemiosmosis was present in the last common ancestor—since the ATP-ase enzymes that work with ion-spanning electronic potentials are found in all domains of life—the presence of a successful metabolic strategy should not be taken as proof of deep history, especially in light of the apparent ease by which genes can be horizontally transferred between organisms. It is possible that none of these three known mechanisms of energy conservation were operative at the OoL; an in-depth assessment of this claim needs to be made, and possible alternatives suggested.

Other basics yet to be addressed include the following: How stable were the first biomolecules, and in what fraction of contemporary values were these first biomolecules present? How did the first organisms accommodate the continual refreshment of unstable molecules within their metabolisms? From an ecological perspective, were early organisms inefficient (requiring large amounts of substrate to survive) or perhaps drastically different? Asked in another way, did the OoL occur in an environment with abundant nutrients and energy where life could exist and evolve in a “wasteful” state, or were the first organisms very efficient and able to make a living in very low energy regimes, perhaps with restricted access to nutrients?

At what level can we begin to generalize about the constitution and organization of a cell? Current biology is revealing that, even within a collection of clonal (genetically identical) microbial cells, each cell has the potential to exist in a different state of gene expression and metabolic state.

Each cell is a unique expression of the potentiality of that organismal type. As measurements become better, some average- (population-) level measurements may need to be replaced or at least amended with data from single-cell measurements.

In what ways can OoL researchers generalize biological properties? At the level of populations or among individuals? And in what ways can OoL researchers conduct measurements on individuals within a population in a manner that would allow the identification of possibly successful cellular states, metabolic, and evolutionary trajectories (compare with Fig. 4)?

Progress may be made through the study of modern environments that are potential analogues of the Archean, for example, hot springs. In addition, a major goal will be laboratory studies that aim to define plausible sets of early catalysts. These catalysts may have been operative to enable otherwise very slow chemical reactions. Determining how these catalysts may function as energy converters, facilitating something similar to the energy conservation pathways we find in extant biology, may be instructive.

Finally, simply learning more about contemporary biology and its operation has the potential to substantially revise thinking of the bioenergetics operative at the OoL. In the same way that a review of A-Life contributions to the OoL problem would be valuable, biological scientists should be encouraged to condense and articulate knowledge of contemporary biological metabolisms and mechanisms for the benefit of, and utilization by, researchers outside the field.

**Making life in the laboratory**. This remains a critical challenge as part of the three approaches to the OoL—historical, synthetic, and universal. We *assume* that it is possible to make life artificially, but it is only recently that studies have begun to appear that explicitly works toward this goal. It is difficult to assess how much progress has been made. The source of the difficulty in achieving this synthesis is the subject of considerable, and considerably contradictory, speculation.

It is possible that our search strategies for detecting the spontaneous development of lifelike processes are hampered by conceptual and analytical impediments. The classic Miller-Urey experiment demonstrated the production of potential building blocks for terrestrial biology, but after ∼60 years it remains unclear how the types of small organic compounds such experiments provide self-assemble, or can be assembled, into living systems.

Historically, efforts to understand the OoL have centered on reactions and compounds that operate in modern biology rather than undirected “coaxing” of systems toward a living state, or analysis of complex mixtures for lifelike processes. This is likely to change in the near future.

**Guiding the next generation of OoL scientists**. A useful way to bring focus to the question of which areas are of near-term importance for the OoL is to consider what we (individually or as a community) would recommend the next generation of students of OoL science study.

It is strongly suggested that the outcomes of this workshop be used to populate an online resource as a first step in this direction.

### 2.2. EON Roadmap

The above set of statements and aspirational goals for OoL research also represent a good set of guidelines for the activities of EON over the next 3 years.

In particular, it is clear that EON can play a key role in this field by supporting a number of specific areas that have been identified for improving and advancing OoL science:

• EON should seek ways to encourage and facilitate communication and collaboration between the fields of A-Life (*i.e.*, theoretical, computational, and robotics work on living systems) and chemical and biological approaches to the OoL, integrated with the research focus of Earth and solar system exploration communities.
• EON should work toward ensuring that ELSI supports multidisciplinary research into the OoL.
• EON should seek the means to encourage and enable innovative and high-profile efforts to produce breakthroughs in OoL studies. Examples may include the organization of “X-Prize”-style competitions that can help build interdisciplinary collaboration and attract new ideas and funding sources to OoL science.
• EON should host an online hub of OoL resources, including review material and a “living” repository of source material and data.
• EON should aspire to provide the field with quantitative evidence for the necessity and efficacy of OoL research (*e.g.*, the outcomes of cross-disciplinary interaction, open-access data). This resource can be used to encourage funding sources to support OoL research.

### 2.3. Relationship to NASA Astrobiology Roadmap and Strategy documents and the European AstRoMap

The NASA Astrobiology Roadmap (Des Marais *et al.* 2003, 2008) and the forthcoming NASA Astrobiology Strategy document (2015) are exemplars of collective opinion, balance, and scientific detail. As such, these have served, and will continue to serve, as invaluable reference points and guides for NASA and NASA-supported communities as well as all other investigative efforts into the nature of life in the Universe. The European AstRoMap consultation project (Horneck *et al.*, 2015) also represents a very significant portion of the landscape of astrobiology research and aspirations.

There are major overlaps between the overarching goals of finding evidence for extraterrestrial life and understanding the mechanisms of life's origins on Earth or elsewhere. Additionally, seeking Earth-analog exoplanets offers the very real prospect of obtaining data proxies to address questions about the geochemical and thermodynamical state of early Earth and the OoL.

The present document should be read as a complementary reference to the NASA and AstRoMap astrobiology documents, one that does not represent consensus opinion, but an effort to capture a number of critical issues and proposals for making progress in understanding the OoL. In particular, we have given ourselves the freedom to present opinions and positions on the ways in which OoL research communities might better work together and to present high-level conceptual statements on the nature of understanding the OoL.

## Appendix I

The Earth-Life Science Institute at the Tokyo Institute of Technology was launched in December 2012 as part of the World Premier International Research Center Initiative (WPI) of the Ministry of Education, Culture, Sports, Science and Technology (MEXT). The WPI grant is awarded to institutes with a research and administrative vision to become globally competitive centers that can attract the best scientists from around the world to Japan.

The Earth-Life Science Institute's research mission is to elucidate how Earth formed and how its early environment allowed for the origin of life and its subsequent evolution. What distinguishes ELSI from other institutes studying the OoL is its emphasis on placing the study in the specific context of early Earth.

The ELSI Origins Network (EON) was created to form a global interdisciplinary network, centered at ELSI, for research into the OoL. Its goal is to bring together existing ideas from different sciences to shape each other's development and create a collaborative research community with global vision, which can recognize and ask the next generation of questions. EON is designed to support ELSI's goal to be a worldwide destination for leading-edge research in all aspects of the OoL and to internationalize research and higher education in Japan. EON is funded by a generous grant from the John Templeton Foundation.

## Appendix II

Summary of responses to a questionnaire sent to workshop participants prior to meeting, which are separated into broad topical areas. Participants were asked to provide examples of the questions or topics they considered most interesting, urgent, and important for OoL science in the near and mid term.

**Early Earth environments, planets (solar system), exoplanets**

• What was the chronology of OoL events on early Earth?
• What were the couplings between the planetary chemical/thermodynamic environment and OoL factors such as early metabolism?
• How does the emergence of key chemical systems take place from disordered states?
• How can OoL hypotheses be constrained with geological/astronomical data?
• Are there habitable environments beyond Earth?
• Is there life beyond Earth?
• Is life a universal phenomenon?

**OoL science “social engineering”**

• Local and global fragmentation of OoL science is an exceptional challenge, needing harmonization and synthesis.
• Recognition of the need for common dialogue and a cooperative community—enabling that communication in an environment of limited institutional support.
• Can we identify broad questions that can be answered independently of each other and hypotheses that can be tested independently from detailed scenarios?
• What is the common framework for chemical specifics to inform/be informed by dynamical generalities?
• Is there a way to combine the many proposed/studied reaction networks in models and experiments to test their compatibility and relation to hypotheses?

**RNA specifics**

• What are the links between RNA and the geophysics/geochemistry of Early Earth?
• How does proto-metabolism (abiotic autocatalytic cycles) connect to the putative RNA world?
• Is there empirical evidence for a diversity of abiotic autocatalytic cycles other than, for example, the classic formose reaction?
• Were the OoL the result of selection within a vast chemo-diversity?
• Were the OoL the result of exploration of a chemical landscape of relatively few “correct” components?
• What is the functional integration between compartments (*e.g.*, lipid vesicles), RNA polymers, and small sets of reactive molecules?
• What is the path for prebiotic RNA monomer synthesis? Is there one?

**Building life**

• Can we make new life (synthetic or artificial) and replace the OoL with A-Life and design a roadmap to then solve the OoL?
• Can we quantify “aliveness”?
• Can we create minimal RNA life in the laboratory?
• Can A-Life escape the limitations of terrestrial biology?
• Can we reconstruct subsets of living states?
• Can we select and characterize a replicase composed of alternate biopolymers and test their fitness?
• Is it possible to build semisynthetic organisms with nonstandard DNA?
• Can we build an automated system to explore chemical inheritance and variation?

**Prebiotics, precursors, early bioenergetics**

• How can we improve current dogma regarding prebiotic plausibility and defining life?
• How does life's information-carrying system emerge?
• Is it possible to identify realistic autocatalytic cycles and systems of cooperation between metabolism, “genes,” and compartments?
• Can we develop with precision knowledge of early Earth conditions and organic matter sources and availability?
• How were critical metabolisms autochthonously generated/organized on Hadean Earth?
• How did chemistry go from order to chaos, and how do we model this?
• How can we perform exhaustive characterization of common minerals as prebiotic catalysts?
• Were the first energy transducers (ion pumps, electron bifurcation, phosphorylation complexes) different than today's, or was there preexisting functionality?
• Are there other ways to harness environmental chemical potentials?
• Are there simple ways to couple molecular organization to dissipation of chemical potentials?

**Big thinking and evolution**

• Is life inevitable? Which properties are universal?
• What is the role of death and extinction in emergence of life?
• Are there new ways to characterize stages of life's history as information storage, processing, and transmission?
• How is the level of organization related to selection and evolutionary dynamics?
• Were cognition and consciousness inevitable?

## Supplementary Materials

Supplementary materials can be found at http://eon.elsi.jp/solr-whitepaper-sm/

• Insights 1: Perspectives
• Insights 2: Chemistry & Origins
• Insights 3: Experiment & Observation
• Insights 4: Evolution, Complexity & Computation

## Abbreviations Used

A-Life: artificial life
ELSI: Earth-Life Science Institute
EON: ELSI Origins Network
ISSOL: International Society for the Study of the Origin of Life
LUCA: last universal common ancestor
OoL: origins of life
WPI: World Premier International Research Center Initiative
